# Mechanical Performance and Applications of CNTs Reinforced Polymer Composites—A Review

**DOI:** 10.3390/nano11092186

**Published:** 2021-08-26

**Authors:** N. M. Nurazzi, F. A. Sabaruddin, M. M. Harussani, S. H. Kamarudin, M. Rayung, M. R. M. Asyraf, H. A. Aisyah, M. N. F. Norrrahim, R. A. Ilyas, N. Abdullah, E. S. Zainudin, S. M. Sapuan, A. Khalina

**Affiliations:** 1 Institute of Tropical Forestry and Forest Products (INTROP), Universiti Putra Malaysia (UPM), Serdang 43400, Malaysia; mohd.nurazzi@gmail.com (N.M.N.); atiyah88@gmail.com (F.A.S.); edisyam@upm.edu.my (E.S.Z.); sapuan@upm.edu.my (S.M.S.); 2 Centre for Defence Foundation Studies, Universiti Pertahanan Nasional Malaysia (UPNM), Kem Perdana Sungai Besi, Kuala Lumpur 57000, Malaysia; 3 Advanced Engineering Materials and Composites (AEMC), Department of Mechanical and Manufacturing Engineering, Universiti Putra Malaysia (UPM), Serdang 43400, Malaysia; mmharussani17@gmail.com; 4 Faculty of Applied Sciences, School of Industrial Technology, Universiti Teknologi MARA (UiTM), Shah Alam 40450, Malaysia; sitihasnahkam@uitm.edu.my; 5 Faculty of Science, Universiti Putra Malaysia (UPM), Serdang 43400, Malaysia; marwahrayung@yahoo.com; 6 Department of Aerospace Engineering, Universiti Putra Malaysia (UPM), Serdang 43400, Malaysia; asyrafriz96@gmail.com; 7 Department of Mechanical and Manufacturing Engineering, Faculty of Engineering, Universiti Putra Malaysia (UPM), Serdang 43400, Malaysia; 8 Research Centre for Chemical Defence, Universiti Pertahanan Nasional Malaysia (UPNM), Kem Perdana Sungai Besi, Kuala Lumpur 57000, Malaysia; 9 Faculty of Engineering, School of Chemical and Energy Engineering, Universiti Teknologi Malaysia (UTM), Skudai 81310, Malaysia; 10 Centre for Advanced Composite Materials (CACM), Universiti Teknologi Malaysia (UTM), Skudai 81310, Malaysia

**Keywords:** CNTs, MWCNTs, SWCNTs, polymer composite, mechanical performance

## Abstract

Developments in the synthesis and scalable manufacturing of carbon nanomaterials like carbon nanotubes (CNTs) have been widely used in the polymer material industry over the last few decades, resulting in a series of fascinating multifunctional composites used in fields ranging from portable electronic devices, entertainment and sports to the military, aerospace, and automotive sectors. CNTs offer good thermal and electrical properties, as well as a low density and a high Young’s modulus, making them suitable nanofillers for polymer composites. As mechanical reinforcements for structural applications CNTs are unique due to their nano-dimensions and size, as well as their incredible strength. Although a large number of studies have been conducted on these novel materials, there have only been a few reviews published on their mechanical performance in polymer composites. As a result, in this review we have covered some of the key application factors as well as the mechanical properties of CNTs-reinforced polymer composites. Finally, the potential uses of CNTs hybridised with polymer composites reinforced with natural fibres such as kenaf fibre, oil palm empty fruit bunch (OPEFB) fibre, bamboo fibre, and sugar palm fibre have been highlighted.

## 1. Introduction

CNTs are cylindrical molecules made up of hexagonally arranged hybridised carbon atoms. Carbon nanotubes are formed from micrometre-scale graphene sheets folded into nanoscale cylinders and topped with spherical fullerenes. Due to the presence of delocalised electrons in the z-axis, CNTs have distinct electrical properties. CNTs are classified according to their wall thickness into single-wall carbon nanotubes (SWCNTs) and multiwall carbon nanotubes (MWCNTs). MWCNTs are multilayered rolled graphene sheets, whereas SWCNTs are nanocylinders constructed from a single graphene sheet. The van der Waals force between CNTs and the weak interplanar interactions of graphene sheets (highly polarised -electron clouds in CNTs) firmly bind CNTs in nature. As a result, the aggregation and solvent chemistry of CNTs nanomaterials regulate their size, shape, and surface area [1,2]. CNTs are now only used as reinforcements in polymer matrices. Nanotubes have outstanding mechanical and physical properties, making them ideal building blocks for high-performance multifibres and composites [3]. Because of their superior mechanical properties and high aspect ratio, CNTs have long been considered a desirable filler for polymer composites [4]. As shown in Table 1, CNT-reinforced polymer composites were first used commercially in the 1980s and have since risen in popularity as a high-performance material in the aerospace, automotive, sports, biomedical, and electronics industries due to their high specific stiffness, strength-to-weight ratio, low thermal expansion coefficient, and high thermal conductivity. Aside from that, CNTs are broadly used as sensing materials in chemical and biosensor applications [5,6].

**Figure 1:**
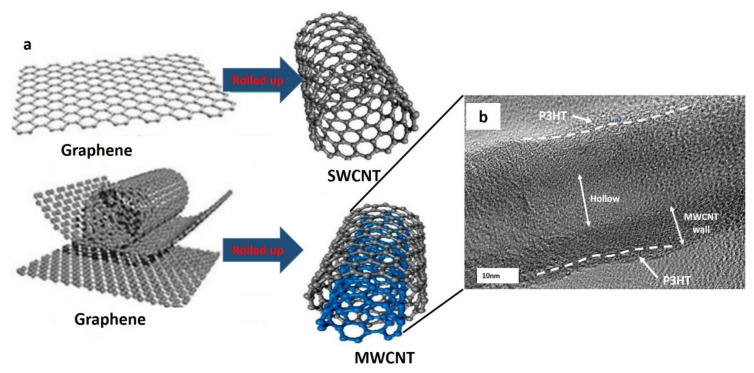
(**a**) Schematic diagram of SWCNT and MWCNT (reproduced from [35]) and (**b**) the MWCNT wrapped with poly(3-hexylthiophene). Reproduced from [37].

Even so, agglomeration and restricted dispersion in the polymer matrix, as well as the van der Waals force between CNTs and weak interplanar interactions of graphene sheets (highly polarised π-electron clouds in CNTs) firmly bind CNTs in nature, making production of advanced composites with CNTs as reinforcement difficult. As a consequence, the size, shape, and surface area of CNT nanomaterials are controlled by aggregation and solvent chemistry. Thus, in the use of carbon-based nanomaterials, overcoming aggregation is critical. When compared to other carbon compounds such as graphite and fullerene, CNTs are hydrophobic and electrically conductive by nature, and they have a large surface area. The large surface area of CNTs results in a high viscosity of the nanotube/epoxy combination when fabricating composites with a high nanotube loading level, making nanotube dispersion difficult. As a consequence, controlled particle size distribution and dispersion are important factors in composite material production. Because the fillers are small, the composites have a high interfacial area [20]. The schematic diagram of SWCNTs and MWCNTs from a rolled graphene sheet is shown in Figure 1 [35,36].

It can be concluded that so far the performance on CNTs in reinforcing polymer matrices has proved inadequate, which several researchers have attributed to two main issues: (1) difficulties in distributing CNTs in polymers, and (2) insufficient bonding of nanotubes with the polymer interface. A substantial amount of research has been conducted on the chemical functionalisation of CNTs to achieve homogeneous dispersion of CNTs in the polymer matrix and high interfacial adhesion between CNTs and polymer matrix [3,16,25]. The results revealed that functionalisation of CNTS surface-enhanced both the adsorption energy, mechanical and electrical characteristics. This happens towards the carbon layer’s margins but can also appear further from the edges if the incorporation sites are related to vacancies. These vacancies and edges might act as adsorption sites, explaining the unusual structures of doped CNTs [33]. Previous studies have shown that controlling CNTs contact improves CNTs-polymer matrix interactions. The degree of interaction between the filler and the polymer modify the mobility of the polymer chain, the degree of curing and the crystallinity of the polymer. The successful integration of interfacial adhesion between CNTs and the relevant polymer matrix could result in significant structural benefits for a variety of applications. As a result, in this brief overview, the mechanical performance and factors influencing the mechanical performance of CNTs reinforced polymer composites and CNTs-reinforced polymer composites for structural applications and their prospects have been discussed. 

## 2. Mechanical Characteristics of CNTs

Dispersion and distribution are key characteristics in the manufacturing of composites. CNTs with good dispersion and homogeneous distribution are favourable for the creation of linked networks [38]. Nevertheless, depending on the type of polymer matrix, a certain degree of agglomeration and a carefully tailored non-homogeneous distribution may lead to segregated structures with excellent mechanical properties.

Overney et al. [39] computed the rigidity of short SWCNTs using ab initio local density calculations to obtain the parameters in a Keating potential. Another study led by Wang and Zhang [40] found out that the effective thickness of SWCNTs should be smaller than 0.142 nm. In this case, Young’s modulus of SWCNTs composites can be attained between 0.65 TPa and 5.5 TPa. Subsequent initial mechanical measurements on MWCNTs created by the arc discharge technique were made. Poncharal et al. [41] generated electromechanical resonant vibrations with moduli ranging from 0.7 TPa to 1.3 TPa. Wong et al. [42] investigated the mechanical characteristics of MWCNTs and found Young’s modulus average value of 1.28 TPa. More importantly, they were able to conduct the initial strength tests, getting an average bending strength of 14 GPa. Table 2 lists the mechanical characteristics of CNTs and other examples of reinforcing materials [36,43,44,45,46,47].

## 3. Factors Influencing the Mechanical Performance of CNTs Reinforced Polymer Composites

The bonding and strength at the interface, in addition to mechanical load transmission from the matrix to the nanotubes surface, all have a major impact on the performance of CNTs-reinforced polymer composites. The mechanism of interfacial load transmission from matrix to nanotubes may be classified into two types: weak van der Waals forces between the polymer matrix and CNTs as reinforcement [48]. Furthermore, one of the most important factors influencing the performance of CNTs-reinforced polymer composites is the dispersion of CNTs in the polymer matrix via physical, functionalisation of CNT surfaces, and their structures [49].

Microcracks can develop during the curing process or due to the wettability of CNTs and matrix interfaces. Microcracking can occur in high modulus resin systems. This is particularly the case at high processing temperatures and low service temperatures, where there is a substantial disparity in thermal expansion between the polymer matrix and the CNT reinforcements. Hence, the use of CNTs as a toughening reinforcement to a polymer resin matrix increases microcracking prevention while compromising performance at elevated temperatures [50]. As a result of the hydrophobic surface regions of the matching micelles surrounding the nanotubes, constraints such as the agglomeration of CNTs arise frequently. Therefore, a detailed understanding of the factors that influence the mechanical properties of CNTs-reinforced polymer composites has been a major consideration.

**Figure 2:**
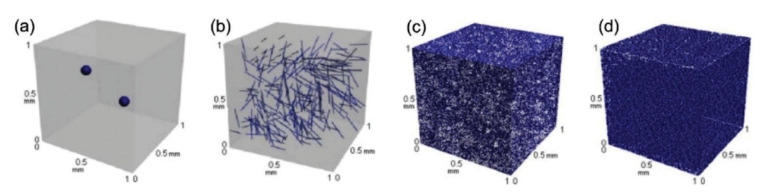
Micro and nano scale distribution of; (**a**) Al_2_O_3_ particles, (**b**) carbon fibers, (**c**) graphene nanoplatelets (GNPs), and (**d**) CNTs. Reproduced with permission from [38]. Copyright Elsevier, 2010.

Aside from the previously mentioned issues of dispersion and agglomeration, the aspect ratio of CNTs is an important factor in the longitudinal elastic modulus. CNTs have a high aspect ratio in general, but their ultimate performance in a polymer composite is dependent on the type of polymer matrix used. Arash et al. [51] investigated the influences of CNT aspect ratio on Young’s modulus and yield strength of CNTs/polymethyl methacrylate (PMMA). The results revealed an increase in Young’s modulus of PMMA polymer reinforced by CNTs, as well as an increase in the CNT aspect ratio. The diameter of the (5, 5) CNTs reinforcements was 0.68 nm, and the length-to-diameter ratio (L/d) ranged from 7.23 to infinity (∞). The stress transfer between the CNTs and the polymer was then enhanced by increasing the aspect ratio of the CNTs. Finally, the CNTs reinforced polymer composites have high strength and stiffness values. Coleman et al. [52] stated the higher the aspect ratio of CNTs, the higher the stress transfers from the polymer matrix to the dispersed CNTs. This is because the CNTs, which have a high aspect ratio, may lead to an adequate load transmission via interfacial shear stress. As a result, the full strength of CNTs can be utilised. Figure 2 shows the effect of different types of nano-scale particle distribution caused by the exceptionally large surface area of the nanocomposites.

**Figure 3:**
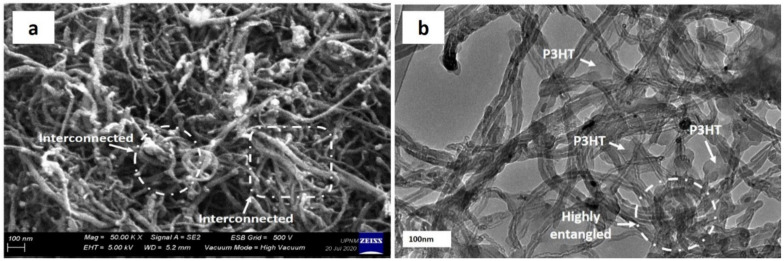
Entangled nature of MWCNT (**a**) under FESEM and (**b**) under HRTEM. Reproduced from [37].

A significant amount of research has been directed toward the fabrication of CNT- reinforced polymer composites for functional and structural applications [53,54,55]. Referring to Ma et al. [38], however, the potential for using CNTs as reinforcements has been greatly restricted due to difficulties associated with entangled CNT dispersion during processing and poor interfacial interaction between CNTs and polymer matrix (Figure 3). The limits to dispersing CNTs differ from those of other conventional fillers such as spherical particles and carbon fibres because CNTs have nanometer-scale properties with aspect ratios greater than 1000, resulting in an exceptionally large surface area. Figure 2 depicts a schematic representation of the 3D distribution of micro-and nanoscale fillers in a polymer matrix, which demonstrates the strong influence of particle size and geometry on the varied distribution behaviour of particles in the matrix. The distribution of micro-scale fillers is homogenous throughout the matrix, as shown in Figure 2a,b, and a differentiation between individual particles in a matrix can be easily created. When CNTs are filled into the same volume of matrix system, however, it is difficult to disperse individual particles equally, as shown in Figure 2c,d. Besides that, a large surface area of nano CNTs means a large interface or interphase area present between the filler and the matrix.

The “interface” in composites is a surface formed by a common boundary of reinforcing fillers and matrix that is in contact and maintains the interfacial bonding in between for load transfer mechanism occurs [56]. The “interphase” is defined as the region with altered chemistry, polymer chain mobility, degree of cure and the crystallinity index that are unique from those of the filler or the matrix. The interphase size of CNTs polymer–matrix composites has been reported to be as large as about 500 nm according to the size and dimension of fillers [57]. Even if the interfacial region is only a few nanometres thick, this would lead to tremendous issues with uniform dispersion and distribution that finally deteriorate the mechanical stability and performance.

Related to MWCNTs and based on Paramsothy [58] in regards to the dispersion of nanotubes, adhesion (contact) at the nanotube–polymer matrix interface, and alignment of nanotubes with the polystyrene (PS) composites, dispersion refers to how individual nanotubes were spread out within the PS matrix after solvent casting and before stretching. It was observed that the dispersion of individual CNTs in composite films of 5 wt.% CNTs content was good but poor (due to the occurrence of CNT clumps) in films of higher (10 wt.% and 30 wt.%) CNTs content. Paramsothy also mentioned that agglomerations or clumps of CNTs are caused by two reasons. Before solvent casting, the purified CNT/PS/toluene suspension was treated with ultrasound (sonicated) for 30 min for homogenisation purposes. The purified CNTs used to form the suspension were mainly in the form of clumps. The first reason was that the ultrasound treatment was only capable of partially separating individual CNTs from the purified CNTs clumps in the suspension. The CNT clumps were made up of individual CNTs that were interlocked with one another. It was possible that the ultrasound treatment did not provide enough energy to overcome completely the interlocking between individual CNTs forming the purified CNT clump. Ultrasonic treatment of the purified CNT/PS/toluene suspension (during its preparation for solvent casting) also resulted in uniform distribution of individual CNTs.

The second reason for the observation of CNT agglomerations in the composite film was that reformation of CNT clumps from individual CNTs (in suspension) was possible in the absence of ultrasound. This was due to the high binding energy between individual CNTs, which resulted from van der Waal’s interactions between the CNTs. The van der Waals interaction among the CNTs was sufficient to physically attract them to one another. The resulting high binding energy among the CNTs was high enough to keep them physically close to one other. Ruoff et al. [59] showed that the van der Waal’s interaction between individual CNTs is sufficient to cause substantial deformation (destruction of the cylindrical symmetry of the CNT) when the CNTs are aligned and adjacent, and that the binding energy between a C60 molecule and a graphite plane is high at 1 eV. Also, no deflocculent was used during the fabrication of the composite film. With insufficient dissolved polymer physically separating the individual CNTs and no use of any deflocculent and ultrasound treatment, it was possible that the van der Waals interaction among the individual CNTs was sufficient to physically attract the individual CNTs to one another and that the resulting binding energy among the individual CNTs physically attracted to one another was high enough to keep them physically close to one another, during solvent casting of composite films of higher CNTs content.

In conclusion, two types of interfaces can be formed in CNT-reinforced polymer composites [58]. In the first type of interface (Type 1 interface), wetting of the CNT by the polymer matrix is good, but the adhesion of the CNT to the polymer matrix is weak. This results in the CNT getting pulled out of the polymer matrix before it can experience fragmentation during composite fracture. In the second type of interface (Type 2 interface), wetting of the CNT by the polymer matrix is good. However, the adhesion of the CNT to the polymer matrix is also good. The Type 2 interface can be further sub-categorised into two forms, Type 2a and Type 2b interfaces. In the Type 2a interface, the good adhesion of the CNT to the polymer matrix results in CNT fragmentation during composite fracture. The polymer matrix is not too ductile such that the interface it shares with the CNT is not held in place during CNT pull-out. In the Type 2b interface, the good adhesion of the CNT to the polymer matrix results in a matrix fracture around the CNT during composite fracture, instead. Following the matrix fracture, the polymer coats the CNT as it is pulled out of the matrix. The polymer matrix is too ductile even after work-hardening such that the interface it shares with the CNT is not held in place during CNT pull-out.

## 4. Mechanical Performance of CNTs Reinforced Polymer Composites

The remarkable success of polymer nanocomposites with the incorporation of CNTs to impart superior performance, particularly in mechanical properties, has been widely reported [4]. Among all the factors that contribute to the excellent properties of the nanocomposites, the individual morphological features of CNTs contribute significantly to determining the performance of the nanocomposites [38]. Their mechanical properties are based on the sp^2^ strength of the C-C bonds of the nanotubes, which is stronger than sp^3^ found in a diamond. This characteristic then makes CNTs good candidates for reinforcement in polymer composites [4]. Meanwhile, the novel properties of CNTs include lightweight, distinct optical characteristics, high aspect ratios and surface area, high mechanical strength, and high thermal and electrical conductivity help to impart excellent properties to the polymer nanocomposites they are incorporated into and make them suitable for a wide range of applications [60].

The mechanical properties of the individual CNTs have also become one of the most vital features that contribute to the outstanding mechanical properties of polymer nanocomposites. Theoretically, CNTs have a Young’s modulus of roughly at 1 TPa, which is approximately five times greater than that of steel, and their tensile strength is in the vicinity of 11 GPa to 100 GPa, which is nearly 100 times higher than that of steel. Because of these characteristics, they are the strongest materials ever invented by mankind [4,61,62,63,64,65]. Similar yet more detailed values have been reported by Vankataraman et al. [59] in their review indicating that the tensile strength of MWCNTs is in the range of 11 GPa to 63 GPa, whereas the elastic modulus for the individual MWCNTs is around 1 TPa. Meanwhile, the tensile strength of SWCNTs is in the vicinity of 22 GPa, whilst Young’s modulus was directly measured and determined to be in the range of 0.79 TPa to 3.6 TPa. The compressive strength of the MWCNTs, on the other hand, was estimated to be in the range of 1 GPa to 150 GPa.

The utilisation of CNTs in polymer nanocomposites relies on their very small size with a high aspect ratio that contributes to the high stiffness and strength of the resulting nanocomposites [4]. Despite their small size, CNTs can also have different dimensions, diameters and lengths that determine the dispersion properties which affect the properties of the nanocomposites. The van der Waals interactions between CNTs also cause agglomeration, resulting in poor dispersion properties. Poor dispersion of CNTs can deteriorate the overall performance of the nanocomposites, especially the mechanical and electrical characteristics. In contrast, homogenous dispersions enable uniform load distributions, thus reducing the load concentration and improving the mechanical properties of the nanocomposites [1,4,66]. Besides, the mechanical properties of the CNTs reinforced polymer composites are also greatly influenced by the type of bonding between the two components, the strength of the interface and the mechanical load transfer from the surrounding to the CNTs filler.

The mechanical characteristics of the CNT-reinforced polymer composites can be further improved via various functionalisation techniques, including physical and chemical functionalisation, to enhance the dispersion capability and improve the CNTs interface. As a result, the interfacial bonding between the CNTs reinforcement and matrix components in the composite system will be improved. Chemical modification, for example, aids in improving the dispersion and solubility of the CNTs in solvents or polymers, thus improving the interaction and reactivity with the matrix via hydrogen bonding [67]. This treatment usually involves the use of strong acids to remove the end caps as well as reduce the length of the CNTs. Oxygenated groups like carboxylic acids, carbonyl and hydroxyl groups were added in the acid treatment to the tube ends and defect sites of the CNTs. These oxygen-containing groups can be further treated with other groups like amides, thiols, etc. [68,69,70,71]. As mentioned by Norizan et al. [1] the mechanical properties of the CNT-reinforced polymer composites can be enhanced by incorporating chemical-functionalised CNTs into the polymer matrix that enables covalent bonding between SWCNTs and MWCNTs. Chemical functionalisation can improve the CNTs and polymer matrix interface, which imparts enhancement to the interfacial strength, thus improving the load transfer mechanism to the CNTs [72].

To date, a variety of polymers have been used to be incorporated with CNTs, including liquid crystalline, water-soluble, thermoplastics, and polymer [66]. The CNTs loading was usually reported to be under 10 wt.% to avoid the agglomeration, which resulted in poor processability and weak properties of the resulting polymer composites [4]. CNTs- reinforced thermoplastics have been commonly reported in the past years based on their positive attributes like high strength, high modulus and low density. Thermoplastic composites offer advantages over thermoset composites in terms of damage tolerance, faster component manufacturing times, indefinite shelf life, better recyclability and an improved work environment [73,74]. Like the aforementioned stress transfer criteria that are required for mechanical improvement, the interfacial adhesion between CNTs and the thermoplastics matrix is unfortunately weak as there is no or little chemical bonding at the CNTs-reinforced thermoplastics interface [75]. To date, various chemical modifications and advanced types of thermoplastics were applied to improve the mechanical properties of the composites. A study by Sattar et al. [65] reported on the mechanical behaviour of PU-reinforced MWCNTs nanocomposites indicating that the most challenging issue with MWCNTs in the matrix is increasing the dispersion of the filler to enhance the load transfer capacity of the composite to the nanotube network. The authors compiled the findings about thermoplastic PU-reinforced MWCNTs and discovered that increasing the nanotube concentration from 0 wt.% to 17.7 wt.% produced a non-monotonic trend, with 9.3 wt.% exhibiting the optimum tensile strength nearly 2.4 times higher than that of neat PU polymer. Meanwhile, Young’s modulus and tensile strength of the sample with the amide-functionalised MWCNTs sample considerably improved with no loss in elongation at break [65,76,77]. In a separate discussion, further improvement in interlaminar shear strength (ILSS) and impact toughness was reported by Liu et al. [73] in mechanical properties of thermoplastic-reinforced composites using hybrid CNTs and commercial carbon fibres in the form of multiscale composites.

Other than thermoplastics, CNTs are added to other polymers to improve the mechanical properties of engineering polymers such as epoxy resins. Among all the types of epoxies available, amine-cured epoxies are considered for the polymer matrix due to their superior engineering performance [78,79]. For example, Uthaman et al. [80] found the addition of CNTs into the epoxy imparted an optimum increment in percentage by 52.9% (flexural strength) and 25.5% (flexural modulus), 29.5% (tensile strength) and 48.1% (tensile) with only 1.5 wt.% addition of CNTs. However, the mechanical properties of the CNT- reinforced epoxy nanocomposites decreased by the addition of 2.0 wt.% of CNTs. In contrast, the mechanical properties of the epoxy-reinforced CNTs were also observed to increase even at high loadings (20 wt.%) CNTs. This finding has been proven by Herceg et al. [81], whereby the addition of the highest loading of CNTs provides a maximum measured Young’s modulus of 5.4 GPa and yield strength of 90 MPa. Although the nanocomposites produced had some porosity (2 vol.%), the modulus and the strength were shown to increase. Better improvement can be achieved with the addition of treated CNTs. For example, Lopes et al. [82] utilised oxidised in thermoset polyurethane elastomer (PU). In that study, addition of only 0.5 wt.% of MWCNT-ox was able to increase the elastic modulus of the PU nanocomposites by 47% with better dispersion as compared to non-oxidised MWCNT.

A comparison study also has been done by Zahid et al. [83] between thermoplastic PU and epoxy thermoset-based composites enhanced with MWCNTs. With the addition of 0.5% MWCNTs, the ILSS showed an improvement of 24.37% in epoxy-based composites and 10.05% in thermoplastic PU composites. Even though the ILSS showed thermoplastic-based composites having lower values compared to thermoset based composites, the thermoplastic PU composites impart inelastic deformation without any trace of brittle fractures. In contrast, the CNTs reinforced epoxy composites showed inelastic deformation followed by brittle fracturing. The brittleness properties, on the other hand, decrease with a higher concentration of MWCNTs due to the crack bridging effect of the CNTs. Table 3 shows the comparison of CNTs and other carbon-based reinforcement materials in polymer composites on mechanical strength.

The amount of CNTs plays a vital role in the mechanical properties of nanocomposites. Yazik et al. [84] investigated the effect of MWCNTs on the mechanical properties of shape memory epoxy (SMEP) nanocomposites. Accordingly, it can be seen that the increment in the tensile properties of nanocomposites could be achieved with the addition of low filler content of CNTs, which is around 0.5 wt.%. Notably, the improvement in tensile strength can be attributed to the high surface area of nanofillers that provide more efficient stress transfer, thus strengthening the materials.

When the CNTs content was increased to 1.5 wt.%, the tensile strength value dropped due to agglomeration that occurred at higher filler content. They found out that the higher MWCNTs content caused poor interfacial adhesion between the polymer and the MWCNTs, which caused aggregations and lumping of the nanofillers [99,100]. This led to a stress concentration area and disrupted the wetting of the nanofillers by epoxy, thus preventing the stress transfer of epoxy to nanomaterials. In addition, the flexural strength of the nanocomposite was also improved significantly by 176% with the addition of 1 wt.% of MWCNTs into the SMEP matrix compared to neat SMEP. The presence of higher dispersion of CNTs inside the SMEP matrix inhibits the mobility of the polymer chain under flexural load [101]. Moreover, the uniform dispersion of CNTs filler provided a uniform distribution of stress, hence, reduced the sites of stress concentrations in the SMEP matrix.

Zakaria et al. [102] analysed the influence of SWCNTs and single-layer graphene (SLG) as reinforcing nanofillers on the mechanical properties of epoxy nanocomposites. Different filler loadings of SWCNTs and SLG (0 wt.%, 0.1 wt.% and 0.5 wt.%) were used in this experimental work. The results showed an improvement in the mechanical performance of epoxy nanocomposites with both SWCNTs and SLG fillers compared with the undoped epoxy matrix. The composites’ tensile strength and modulus increased by 14% and 21%, respectively, when 0.5 wt.% SWCNTs were added, which was attributed to several factors, including cross-linking interactions that enhanced the polymer to nanofiller interactions. Interestingly, the SWCNTs/epoxy nanocomposites showed higher tensile strength and modulus as compared with SLG/epoxy nanocomposites. The tensile strength of SWCNTs-based nanocomposites was higher than that of the SLG-based nanocomposite, as the SWCNTs filler has a high filler length and aspect ratio. SWCNTs-based nanocomposite with 0.5 wt.% SWCNT displayed the highest tensile strength and tensile modulus of 49.07 MPa and 1.70 GPa, respectively, as compared with SLG-based nanocomposite with 0.5 wt.% SLG with 48.01 MPa and 1.62 GPa, respectively. An increment of about 2% and 5% in tensile strength and modulus value of SWCNT-based nanocomposite is higher than that of SLG-based nanocomposite. The enhancement is easily explained by the properties of SWCNTs, which have higher dispersion and different shapes of filler than SLG. In the case of SWCNTs, some of the wire-like structures of the SWCNTs show twists and kinks which could prevent the detachment of the SWCNTs from the epoxy matrix. Meanwhile, for SLG, the crumpled and wrinkled thin film of the SLG structure seemed to detach more easily from the epoxy matrix compared with the SWCNTs structure. Therefore, SWCNTs was able to be dispersed more effectively in the epoxy matrix than SLG. Furthermore, the weak interaction of SLG-based nanocomposites than SWCNT nanocomposites could be because of van der Waals forces acting between the adjacent SLG, resulting in lower tensile strength and modulus value of SLG nanocomposites than SWCNT nanocomposites [103].

Sapiai et al. [104] reported on the mechanical properties of functionalised CNTs added to kenaf-reinforced epoxy composites. The tensile, flexural, and impact properties of the kenaf/epoxy composite were strengthened by 43.30%, 21.10%, and 130%, respectively, when 1 wt.% acid-silane-treated CNTs (ACNTs) were included. The mechanical study indicates that the composite with 1 wt.% acid silane-treated CNTs loading exhibited the best value mechanical performance. With increasing ACNTs filler contents of 0.5 wt.%, 0.75 wt.% and 1.0 wt.%, the ACNTs/kenaf/epoxy composites demonstrate increments of 0.08%, 0.76% and 8.66% in flexural strength compared to the unfilled kenaf composites. It was concluded that acid and silane treatment on CNT surfaces increased the flexural strength and modulus because the acid and silane treatment process aided in functionalising the CNTs surfaces. This is because the existence of the –COOH and Si–OH groups had improved CNTs surfaces by enhancing dispersibility and reducing agglomeration of CNTs in the epoxy matrix. Moreover, the impact strength continued to increase for 0.5%, 0.75% and 1.0% of CNTs kenaf/epoxy composites where the increments observed were about 84.12%, 86.51% and 130%, respectively. The ability of CNTs to absorb more impact energy compared to the epoxy matrix contributes to the remarkable improvement in value in impact strength. Therefore, the toughness of the material could be further improved with more energy absorbed by the material.

A comparison of bamboo/CNT reinforced epoxy hybrid composite and alkali-treated bamboo epoxy composite was conducted by Kushwaha et al. [105]. The functional groups which are formed on the CNTs surface had improved the interfacial bonding between the CNTs and the surrounding matrix. CNTs addition results in an improvement in the interfacial bonding by giving rise to additional sites of mechanical interlocking that facilitate load transfer. The formation of covalent bonds between the CNTs and epoxy resin facilitates load transfer between the CNTs and epoxy matrix and contributes to the improvement in the mechanical properties of the composites. Remarkably, there was a significant increase in impact strength by 84.5% due to the flexibility of the interface molecular chain, resulting in comparatively greater energy absorption.

## 5. Potential Applications of CNTs

Carbon-based nanofillers reinforced polymer composites have gained popularity for a variety of applications due to their superior properties [38]. The varied applications of these polymer nanocomposites rely on the superior properties possessed by the CNTs themselves. Furthermore, the good compatibility of CNTs with polymer matrices has increased the potential of these materials for being used in a variety of advanced applications, such as electronics, automotive, textiles, aerospace, sports equipment, sensors, energy storage devices, filters [4,106,107,108,109,110].

**Figure 4:**
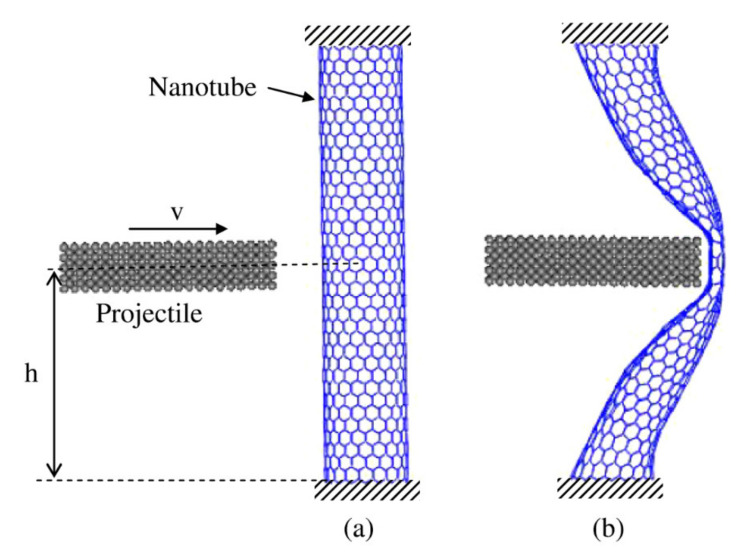
The molecular dynamics model of a CNT subjected to ballistic impact. (**a**) Initial model, (**b**) a deformed (18, 0) nanotube at its maximum energy absorption. Reproduced with permission from [113]. Copyright IOP Publishing, 2007.

Polymer nanocomposites reinforced with CNTs have also been reported as an excellent choice for the fabrication of ballistic armour materials, owing to their outstanding stiffness and strength, large fracture resistance, light density, and high energy absorption, which increases their potential for use in body armour [111]. When a bullet hits body armour, the material’s fibres absorb and distribute the impact energy to subsequent layers so that the bullet does not penetrate through the body armour. However, blunt force trauma or non-penetrating injuries may still be caused by dissipation forces. The collision and resultant trauma will cause severe damage and injure critical organs, even when the bullet is stopped by the body armour. Thus, a high degree of elastic storage energy should be used as the ideal material for body armour which causes the bullet to be rebuffed or deflected. According to Benzait et al. [111], polymer-reinforced CNTs are an excellent choice for ballistic armour materials due to their remarkable stiffness and strength, low density, large fracture resistance and high energy absorption. The findings reported by Hanif et al. [112] on the influence of CNTS inclusion on the fracture and ballistic resistance in twaron/epoxy composite panels support this statement. The study revealed that with only 1 wt.% addition of MWCNTs, they were able to significantly improve fracture toughness and ballistic resistance with increased impact energy absorption value. Another study conducted by Mylvaganam and Zhang [113] found the highest ballistic resistance capacity of a CNTs is when the bullet hits its centre and a larger tube withstands a higher bullet speed. They also fabricated a body armour made of six layers of 100 μm nanotube yarn with a thickness of 600 μmin that could bounce off a bullet with the muzzle energy of 320 J. A study led by Han and Elliott [114] conducted a study on classical molecular dynamics simulations of model polymer/CNT composites constructed by embedding a single wall (10, 10) CNT into two different amorphous polymers matrices. They found out that it is possible to use CNTs to mechanically reinforce an appropriate polymer matrix, especially in the longitudinal direction of the nanotube. Other literature reports on dynamic molecular simulation studies conducted of CNTs-reinforced polymer composites are those of Zhang and Shen [115], Chang [116], Ni et al. [117], Shen et al. [118], Fan et al. [119] and Lin et al. [120]. Figure 4 displays the molecular dynamics model of a CNT subjected to ballistic impact.

**Figure 5:**
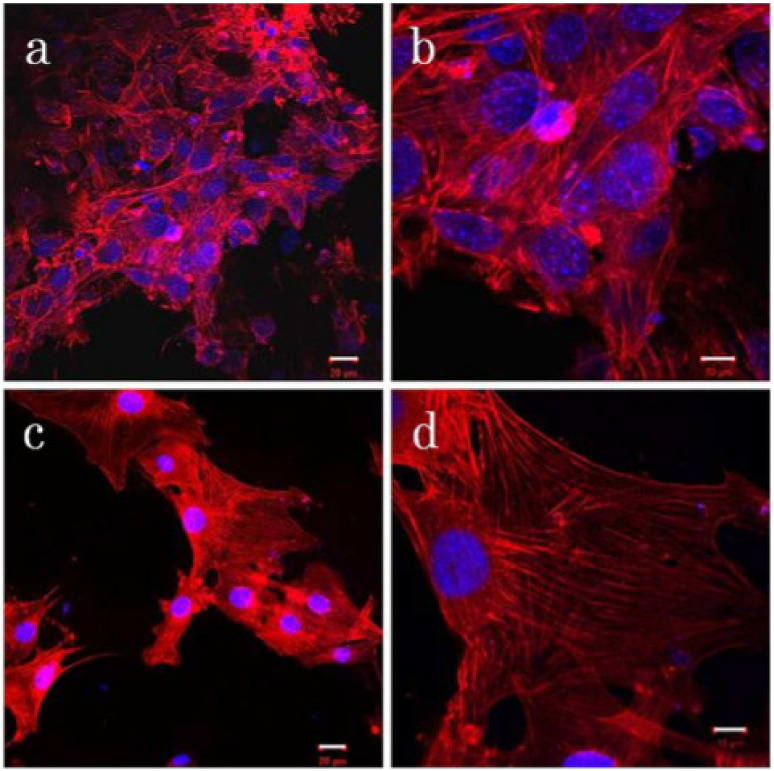
Fluorescence photomicrographs of cell cultures on (**a**,**b**) PET reinforced collagen sheets and (**c**,**d**) MWCNTs blocks. Reproduced from [122].

Recently, the development of CNTs-based nanocomposites for biomedical applications has been reported, particularly in tissue engineering and drug delivery [60]. The unique graphitic structure and the superior performance of CNTs for their mechanical, electrical, optical and biological characteristics have allowed them to be used in biomedical field applications like gene/drug delivery and tissue engineering. According to Huang et al.’s review paper [121], researchers have documented the use of CNTs as substrates for neuronal tissue engineering because CNTs can assist neuron attachment, allow the generation of longer and more elaborate neuritis, as well as promote cell differentiation. CNTs- based polymer composites are also employed in the formation of bone scaffolding materials. Tanaka et al. [122], for instance, employed the 3D block structure of CNTs to study their efficacy as scaffold materials for bone repair. They found the CNTs scaffolds for cell adhesion as compared to PET reinforced collagen scaffolds with good osteogenesis behaviour, as shown in Figure 5. Other than that, CNTs have also been considered to serve as drug and gene delivery carriers. Their easy surface functionalisation has prompted their use to deliver different genes, including plasmid DNA (PDNA), micro-RNA, and small infecting RNA as gene delivery vectors for various diseases for instance, cancers [123].

**Figure 6:**
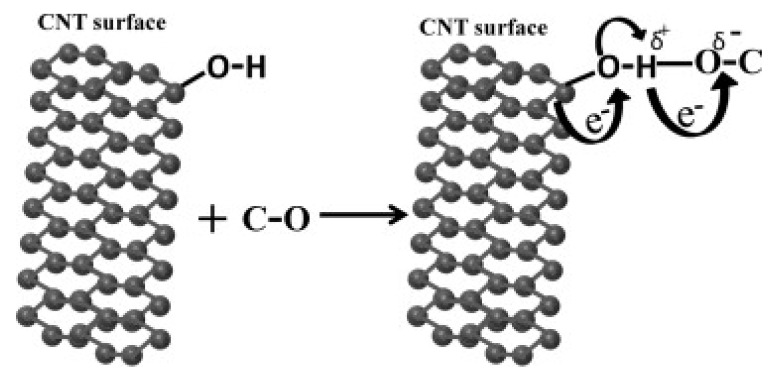
Adsorption of CO gas molecule on the hydroxyl modified CNTs. Reproduced from [158].

CNTs are ideal materials for gas sensors due to their inherent characteristics such as high porosity and high specific surface area [124]. The main concern with the burning of fossil fuels is toxic gas emissions. The identification of these gases is crucial for saving the environment and humans from the dangers posed by the gases generated by the combustion of fossil fuels. In consideration of gas sensor applications, the physical and chemical characteristics of CNTs were discussed critically in many works [125,126,127,128,129,130]. Some metallic nanoparticles such as Pd, Pt, Au, Ag, Rh, Pb, and Sn have catalytic properties and allow for the specific binding of gas molecules. Variations in the barrier potential of CNTs-metal contact or CNTs-CNTs junctions cause changes in CNT resistance in defect free CNTs. The gases released during the combustion of fossil fuels, such as CO_2_ [131,132,133], CO [134,135,136,137], SO_2_ [138,139,140,141,142,143,144,145,146,147], NO_2_ [136,148,149,150,151,152,153,154,155], and NO [156,157], adsorb on the CNT surface either physically or chemically. Figure 6 shows the bonding behaviour and charge transfer between CNTs and the molecules of C–O. The H atom of functionalised O-H modified CNTs bonds to the electronegative oxygen of carbon monoxide. During the purification procedures, OH groups are attached to CNTs to remove the contaminants.

Several other advanced applications of CNTs-based composites have also been reported in the automotive, aerospace, marine and sporting goods industries. The potential of these materials to be applied in the aforementioned advanced applications can be improved by hybridising the CNTs with other materials, including natural fibres [45,110,159,160,161,162,163]. For example, CNT-polymer composites have been applied to the production of vehicles with the goal of reducing the weight of the body parts, which allows the vehicle to have lower fuel consumption and minimise global warming effects by reducing carbon dioxide emissions. Yang et al. [164] discovered that a 25% reduction in vehicle weight can reduce up to 250 million barrels of crude per year. Therefore, many car manufacturers have employed CNTs-based composites in vehicle parts, including trunk lids, car seats, dashboard coverings, and roofs.

The CNTs-based polymer composite applications in the automobile industry include advancements in current technology such as in body components, electrical systems, and engine parts. The addition of CNTs reinforced with fibreglass in epoxy composites could increase the strength and impact energy by 60% and 30%, respectively. This would subsequently contribute to a reduction in fuel consumption and greenhouse gas emissions by 16% and 26% [165]. The next use of CNTs is for a bendable or flexible battery that is produced by applying an ink-coated sheet of paper or plastic to a CNTs/Ag nanowire-infused substrate. This battery is adaptable to many vehicle applications because of its potential use in portable and wearable electronics. CNTs offer many potential benefits due to their advantages, including high electrical conductivity, the unique structure of 1D nanoscale, suitable surface chemical properties, high degree of graphitisation, and superior electrical performance, which may play a key role in the development of high-performance flexible batteries [166]. CNTs are also found in vehicles tires, which are surrounded by a matrix of polybutadiene and styrene-butadiene rubber (SBR), which are employed as colouring and reinforcing agents during tire production. Andrews et al. [167] used CNTs thin-film transistors (CNTs-TFT) as a tool for sensing environmental pressure on the tire. Shao et al. [168] found that CNTs-filled in passenger tire tread compounds have been shown to offer better handling and traction properties, making them ideal for racing and sports vehicle tires.

Extensive research on the potential of CNTs in the aerospace industry has been conducted to produce composite materials as very high strength and durability aircraft components. The incorporation of CNTs into complicated aircraft designs creates lightweight, minimal cost materials for engines and components, as well as reduced waste in the production processes [169]. The vibration damping factors of the polymer nanocomposites with CNTs sheet reinforcement were found to be significantly reduced, with an enhancement in mechanical, electrical, and thermal characteristics of the MWCNTs composite for structural aerospace applications [170]. Venkatesan et al. [171] observed reductions in coefficient of friction of wear properties in glass fibre hybrid CNT-based composites as a result of the combination of polymer resins in an aluminium–titanium–magnesium matrix, which represent an alternative for passive thermal coverings. A study by Kwon et al. [172] successfully fabricated well-dispersed CNT-based aluminium matrix composites using ball milling and hot pressing processes. In this work, they discovered that the hardness of the CNT-Al composites was significantly enhanced about seven times compared to pure aluminium. Another interesting study in the aerospace application was performed by Laurenzi et al. [173] emphasising the effect of varying loadings of SWCNT and GO nanoplatelets on the equivalent dose received by the nanocomposites in various radiation fields in space, as well as numerical analysis that showed how atoms in nanomaterial formations were arranged. It was noted that CNTs and GNPs suspended in an epoxy matrix decreased the impact damage produced by micrometeoroid orbital debris (MMOD), and the loading and radiation shielding were improved with the addition of GO fillers. Thus, CNTs and GNPs were used to make sensors for aerospace applications. It also recorded a reduction of about 18% in weight and 2.4% in neutron production of radiation shielding spacesuit applications produced from improved MWCNT embedded in PMMA matrix [174]. Furthermore, the electromagnetic interference (EMI) shielding efficacy of MWCNT/polypropylene composites increased as CNTs content and shielding plate thickness increased, demonstrating the efficiency of the CNTs nanocomposites as a heat-absorbing media in the aerospace industry [175].

## 6. Environmental, Health, and Safety Concerns in Utilisation of CNTs

The toxicity, health and safety concerns of CNTs are influenced by several factors, such as aspect ratio, length, surface area, degree of aggregation, purity, and concentration or loading [176]. According to Donaldson et al. [177], repeated exposure of CNTs over a long period may contribute to some common diseases associated with asbestos exposure that has a high mortality burden, triggering global pandemics in the 20th century. Chronic inflammation, formation of granuloma, and fibrosis are among those common anticipated diseases from CNTs persistence [178].

### 6.1. Aspect Ratio

The fact that CNTs have smaller aspect ratios than other reinforcing fillers like carbon fibres, carbon blacks, and clay, means they have better compatibility with the polymer matrix, due to the formation of uniformity of CNTs in the composite’s matrix. Other than uniformity concerns, an international standard regarding the allowance of inhalation of respirable fibre into the lungs has been highlighted by the World Health Organisation (WHO). Only CNTs with a length greater than 5 µm and a diameter of less than 3 µm with a minimum aspect ratio of 3:1 are accepted to be inhaled into the lungs. Otherwise, the large aspect ratio of CNTs affects their behaviour in which they are more difficult to be engulfed and cleared off from the site of deposition of targeted organs due to their propensity to aggregate and form bundle structures of CNTs [179]. Consequently, prolonged exposure to bundle pathogenic CNTs causes bronchogenic carcinoma, mesothelioma, asbestosis, pleural fibrosis, and pleural plaque which cause the pleural pathologies in the end [177].

The standard of occupational exposure limit values (OELs) has been established as legislation applicable to handling nanomaterials to ensure health and safety protection during exposure to CNTs to the environment. Table 4 presents the information OELs for nanomaterials [180]. In detailed findings, the United States of America-National Institute for Occupational Health and Safety (NIOSH) recommends OELs for CNTs to be in the range of 1 to 50 µg/m^3^ as an 8 hours’ time weighted average-TWA µg/m^3^ [181].

### 6.2. Length

The relationships between the lengths of CNTs like MWCNTs in connection to pulmonary fibrosis have been investigated [44]. The result shows that long MWCNTs have higher detrimental pulmonary effects than MWCNTs. However, long CNTs cannot pass through the stomata and are retained, thus causing inflammation diseases. According to Poland et al. [182], the effect of short CNTs (<15 µm) through direct instillation of fibre into the pleural cavity of mice was investigated as compared to long CNTs with a length of 5 µm to 20 µm. With the long type of CNTs (>15 µm), significant inflammation leading to various cell damages could happen due to the disability of the long CNTs to be effectively engulfed by gathering macrophages, resulting in frustrated phagocytosis.

### 6.3. Surface Area

CNTs surface area has been pointed out as another critical aspect as a factor of toxicity. Kim et al. [183] investigated the toxicity of a nanomaterial to be highly affected by its physical properties, such as size distribution and surface area reactivity of particles. In bronchoalveolar lavage fluid (BALF) cell analysis, MWCNTs are found to induce more severe acute inflammatory cell recruitment than acid-treated multiwalled carbon nanotubes (tMWCNTs). This is due to the reduction in the size of the nanoscale increasing the surface area ratio of the materials. As a result, the potential for damage has increased, but this was not possible while they were in larger forms [184].

Considering the higher surface area and lower density of CNTs characteristics, these toxicant particles provide a higher contact area with biological structures, including gas exchanges across alveolar walls in which the total surface area of the alveoli may exceed 100 m^2^. As demonstrated for high aspect ratio materials, this high surface area of CNTs often leads to pronounced biological activity [185]. In comparison with MWCNT, the toxicity of SWCNT was found to be 8.5-fold more fibrogenic than MWCNTs per microgram of dose, causing inflammation in the lungs, resulting in respiratory failure. Dong and Ma (2014) have shown that the lighter and larger surface area of SWCNT than MWCNT are the two factors contributing to the higher level of toxicity of SWCNT on an equal weight basis [179].

Volume per specific surface area is among the complementary criterion for exposure assessment and identification of potential risk [186]. Therefore, the surface of CNT requires modification to alter its toxic responses. With respect to that, the modification of the surface of CNT has been accomplished through the use of acid treatment. This technique is an effective modification by oxidising CNT to introduce carboxyl and hydroxyl groups on the surface of CNTs, resulting in changes in bioactivity and interaction with other molecules [179]. Carrero et al. [187] revealed that nitrogen-doped MWCNTs showed significantly reduced toxicity as well as better tolerance in exposed mice than pristine MWCNTs. In another study conducted by Taylor et al. [188], a thin film of aluminium oxide (Al_2_O_3_) coated MWCNTs induced lower fibrosis in mice as compared with pristine MWCNTs exposures.

### 6.4. Concentration

A compilation of several sets of literature of cell viability to interaction with different types and concentrations of functionalised SWCNTs (f-SWCNTs) and functionalised MWCNTs (f-MWCNTs) is presented in Table 5. Based on this review, the observation from tests on T-lymphocytes by Bottini et al. [189] found out that a safe dosage value of CNTs is around 40 μg/mL. Further, Bianco et al. [190] discovered death in 50% of HeLa (Henrietta Lacks) cells in culture after 6 h of incubation with increasing doses of f-SWCNTs and f-MWCNTs at a concentration of 5 mg/mL to 10 mg/mL. CNTs concentration ranging from six orders of magnitude (from 5 mg CNT/mL to 10 mg) could imply toxicity and resistance within the biological system [191]. Another related study was conducted on the negligible toxicity in the main organs (liver, lung and spleen) of exposed mice after intravenous exposure to CNTs of increasing concentration through constant malondialdehyde (MDA) levels for three months [192]. Results from the long-term accumulation and toxicity of intravenously injected SWCNTs indicate that slight inflammation and inflammatory cell infiltration occurred in the lungs. However, serum immunological indicators (CH 50 level and TNF-α level) remain unchanged and no apoptosis was found in the main organs.

Patlolla et al. [201] investigated hepatotoxicity and oxidative stress in male Swiss-Webster mice exposed to functionalised MWCNTs (f-MWCNTs) at different dosages. The investigation aims to assess the effects, after intraperitoneal (ip) injection, of f-MWCNT on various hepatotoxicity and oxidative stress biomarkers. The mice were dosed at 0.25 mg/kg/day, 0.5 mg/kg/day, and 0.75 mg/kg/day for 5 days. The results show a short-term and high toxicity in mice exposed to f-MWCNTs were recorded and ROS induction, increase in the level of LHP, serum biochemical changes, and damage to the liver tissue were observed. The result indicates that the f-MWCNT induces hepatotoxicity. The authors also suggested that the high toxicity of f-MWCNTs does not imply that they should be banned for biomedical applications, but rather improving the dispersion and excretion of MWCNTs by further chemical modification is essential for safe occupational and environmental exposure to nanomaterials.

## 7. Conclusions and Future Perspectives

In this review, the mechanical performance of CNTs-reinforced polymer composites has been discussed. In essence, CNTs have excellent chemical and physical properties that make them ideal and promising reinforcements in polymer composites. Based on existing studies, it has been acknowledged that the mechanical properties of the CNTs polymer composites are influenced by the interactions between the nanofillers and the polymer matrices. The challenge is mainly the tendency of the CNTs to agglomerate, resulting in poor dispersion properties, which can deteriorate the whole performance of the composite structures. Researchers have come up with various methods for distributing and orienting the CNTs. Further, it has been found that dispersing a small amount of filler in the polymer matrix enhances the properties of the composites. Though many excellent CNT composites have been achieved, constant progress is needed to obtain composites with the best performance. Several aspects, such as the number of CNTs used, size of fillers, spatial distribution and orientation, suitable surface modifications on CNTs surface, and methods of fabrications, affect the mechanical properties of the composites. It is crucial to find an optimum balance between these parameters. Therefore, addressing all the concerns raised will be fascinating to study in the forthcoming investigation into utilising the potential of CNTs in polymer composites.

## Figures and Tables

**Table 1:** Shows several examples of CNTs reinforced polymer composites made in the 1980s, organised by fabrication method.

Year	CNTs	Matrix	Fabrication Method	Ref.
1998	MWCNTs	Epoxy	Solution casting–curing	[7]
1999	CNTs	PVA	Solution casting	[8]
2002	MWCNTs	Epoxy	CVD–injection molding	[9]
2002	MWCNTs	PS	Spin-casting	[10]
2003	SWCNTs	Alumina	Spark-plasma sintering	[11]
2003	MWCNTs	Epoxy	Solution-casting	[12]
2004	MWCNTs	P(MMA-co-EMA)	Solution-mixing	[13]
2004	MWCNTs	Nylon 6	Melt compounding	[14]
2005	MWCNTs	PA	In situ polymerization	[15]
2006	MWCNT–NH_2_	Nylon 6	Solution-casting–melt compounding	[16]
2007	MWCNTs	Aluminium	Isostatic pressing–hot extrusion techniques	[17]
2007	SWCNTs	PVC	Film casting	[18]
2007	MWCNTs	PVC	Film casting	[18]
2008	MWCNTs	PMMA	CVD–solvent casting	[19]
2008	MWCNTs	PS	CVD– solvent casting	[19]
2010	MWCNTs	Epoxy	Ultrasonication technique–sputtering	[20]
2010	DWCNTs	Magnesia	In situ polymerization–spark-plasma-sintering	[21]
2010	MWCNTs	PP	Melt mixing–in situ polymerization	[22]
2011	MWCNTs	Epoxy	Chemical functionalization–cast molding	[3]
2013	Dense-CNTs	PP	CVD	[23]
2014	MWCNTs	PVC	Film casting	[24]
2015	Amino-MWCNTs	Epoxy	Direct stirring–resin infusion molding	[25]
2015	MWCNTs	HDPE	Melt-mixing–compression molding	[26]
2016	SWCNTs	Chitosan	Solution-casting	[27]
2016	CNTs	Epoxy	Press cured method	[28]
2017	MWCNTs	Epoxy	EPD	[29]
2018	MWCNTs	PMMA	Chemical functionalization–micro compounding–injection molding	[30]
2019	MWCNTs	Epoxy	Non-destructive synthesis technique	[31]
2020	MWCNTs	Epoxy	Solution-casting–hand lay-up–resin infusion	[32]
2020	MWCNTs	PVC	CVD–ultrasonic dispersion–extrusion	[33]
2020	MWCNTs	PVC	CVD–ultrasonic dispersion–extrusion	[33]
2021	MWCNTs	Epoxy	Resin castings (injection-molding)	[34]

CVD—chemical vapor deposition, EPD—electrophoretic deposition, ESD—electrospray deposition and CF—chemical functionalization, GF—glass fibre, NBCNT—nitrogen-doped bamboo-shaped CNT, PP—polypropylene, DWCNT—double-walled CNT, PMMA—Poly (methyl methacrylate), PVA—poly (vinyl alcohol), PVC—polyvinyl chloride, P(MMA-co-EMA)—copolymer of methyl and ethyl methacrylate.

**Table 2:** Mechanical properties of CNTs with other example of reinforcing materials.

Reinforcement Materials	Young’s Modulus (TPa)	Tensile Strength (GPa)
SWCNTs	0.65 to 5.5	126
MWCNTs	0.2 to 1.0	>63
Monolayer Graphene	1.0	130
Stainless steel	0.186 to 0.214	0.38 to 1.55
Kevlar	0.06 to 0.18	3.6 to 3.8
Diamond	1.22	>60
Aluminium	71	0.65
Glass fibres	72	3
Carbon fibres	300	3
Silicon carbide fibres	450	10
Sugar palm fibre	0.0049	0.00016
Kenaf fibre	0.053	0.00025
Bamboo fibre	0.0011 to 0.0017	0.00014 to 0.00023

**Table 3:** Mechanical properties of various carbon-based as reinforcement materials in polymer composites.

Reinforcement Materials	Matrix	Mechanical Strength					Ref.
		Tensile Strength (MPa)	Flexural Strength (MPa)	Impact Strength (J/m)	Elastic Modulus (GPa)	Hardness (GPa)	
CB	PVC	35 (−34%)	-	-	-	-	[85]
CB	PP	25 (−47%)	-	-	0.25 (−23%)	-	[86]
CB	PP	60 (100%)	68 (70%)	56 (65%)	4.2 (68%)	-	[87]
CB	Epoxy	58 (190%)	90 (125%)	-	2.6 (200%)	-	[88]
CB	Unsaturated polyester	40 (−14%)	72 (−25%)	-	1.3 (80%)	0.17 (17%)	[89]
CB	NBR/EPDM	16.7	-	-	-	-	[90]
Carbon fabric	Epoxy	580	-	-	67.5	-	[91]
MLG	PVC	19 (17%)	-	-	6 (1%)	-	[92]
Graphene	PVC	55 (130%)	-	-	2 (58%)	-	[93]
Graphite	PS	29 (16%)		21 (−28%)			[94]
Graphite	POBDS	NA	42.5 (0%)	-	-	-	[95]
Graphene oxide	PMMA	180 (−18%)	-	-	8 (−33%)	-	[96]
Graphene sheets	PS	40 (60%)	-	-	2.25 (50%)	-	[97]
Graphite	Epoxy	41 (21%)	-	-	3.3 (10%)	-	[98]
Graphene	PVC	140 (8%)	-	-	5.3 (10%)	-	[24]
MWCNTs	PVC	NA	-	-	NA	-	[24]
MWCNTs	Epoxy	-	105 (110%)	-	-	-	[20]
MWCNTs	Epoxy	52.4	-	-	3.23	-	[3]
MWCNTs	Epoxy	85.6 (13%)	121.6 (0.7%)	23.4 (60%)	2.9 (10%)	-	[34]
MWCNTs	Epoxy	720 (16%))	-	-	54 (4%)	-	[31]
CNTs	Epoxy	-	-	-	9 (−18%)	-	[28]
NBCNTs	PVC	29.5 (−5%)	-	-	0.35 (0%)	-	[33]
MWCNTs	PVC	28 (−9%)	-	-	0.3 (−14%)	-	[33]
MWCNTs	P(MMA-co-EMA)	74 (57%)	-	-	2.3 (130%)	-	[13]
MWCNTs	PMMA	25 (0%)	-	-	2 (33%)	-	[19]
MWCNTs	PS	16 (0%)	-	-	1.5 (36%)	-	[19]
MWCNTs	PS	30.6 (36%)			3.4 (122%)		[10]
CNTs	PP	24 (71%)	34 (35%)	155 (34%)	-	-	[23]
CNTs	Epoxy	1300 (24%)	1078 (10%)	-	-	-	[29]
Amino-CNTs	Epoxy	370 (37%)	225 (80%)	-	8 (33.3%)	-	[25]
MWCNTs	Epoxy	535.4 (4%)	-	-	-	-	[32]
MWCNTs	HDPE	-	-	-	4.7 (47%)	0.1 (15%)	[26]
MWCNTs	PP	35 (25%)	-	4 (54%)	0.8 (23%)	-	[22]
MWCNTs	PA	65.9 (8.2%)	-	-	-	-	[15]
MWCNTs	PMMA	60 (20%)		1.3 (−36%)	-	-	[30]
DWCNTs	Magnesia	-	-	-	-	12.2	[21]
CNTs	Epoxy	-	-	-	3.7 (19%)	-	[7]
MWCNTs	Epoxy	6 (500%)	-	-	0.5 (290%)	-	[9]
MWCNTs	Nylon 6	40.3 (124%)	-	-	0.9 (115%)	-	[14]
MWCNTs	Nylon 6	59.3 (70%)	-	-	3.6 (90%)	100 (67%)	[16]
SWCNTs	Alumina	-	-	-	-	16.1 (−21%)	[11]
SWCNTs	Chitosan	-	-	-	8 (25%)	-	[27]
CNTs	Aluminium	520 (33%)			103 (41%)	1.3 (30%)	[17]

CB—carbon black, MLG—multi-layer graphene, CF—carbon fibre, SPS—sugar palm starch, NBR/EPDM—acrylonitrile-butadiene/ethylene-propylene-diene rubber blends, PDMS—polydimethylsiloxane, PS—polystyrene, POBDS—poly (4,4′-oxybis (benzene) disulfide), PA—polyamide 6, NA—non-applicable.

**Table 4:** OELs for nanomaterial handling.

Category	Benchmark Exposure Level
Fibrous, a high aspect ratio insoluble nanomaterial	0.01 fibres/mL
Any nanomaterial that is already classified in its molecular or in its larger particle form a as carcinogenic, mutagenic, reproductive, and sensitizing (CMRS) toxin	0.1 × OEL
Insoluble or poorly soluble nanomaterials not in the fibrous or CMRS categories	0.066 × OEL
Soluble nanomaterials not in the fibrous or CMRS categories	0.5 × OEL

**Table 5:** Compilation literature studies of toxicity cellular and tissue of different concentration and types of CNT.

Types of CNTs	Concentration	Biological System	Toxicity	Ref.
Plasmid DNA-SWCNTs and Plasmid DNA-MWCNTs	10 mg/mL	f-CNTs: HeLa cell lines in vitro	50% survival of HeLa cells	[193]
Pristine SWCNTs	7.5 μg/mL water	SWCNT: Mesothelioma cell line MSTO-211H in vitro	10% decrease in cell proliferation and activity	[194]
RNA-polymer SWCNTs conjugate	1 mg/mL	MCF-7 breast cancer cells in vitro	No significant cell damage	[195]
Pristine MWCNTs	40 μg/mL	Human T lymphocytes in vitro	No toxicity on human T lymphocytes	[189]
Ammonium chloride-SWCNTs, and poly(ethylene glycol)-SWCNTs	10 μg/mL water	Macrophages, B and T lymphocytes from BALB/c mice spleen and lymph nodes in vitro	5% decrease in viability of B lymphocytes, but no adverse effects on T lymphocytes and macrophages	[196]
125I-SWCNT-OH	1.5 μg/mouse	Intraperitoneal, intravenous, subcutaneous, in male KM mice in vivo	Accumulate in bone, but good biocompatibility	[197]
Streptavidin-SWCNT	0.025 mg/mL	HL60 and Jurkat cells in vitro	No adverse effects	[198]
SWCNTs dispersed in DMEM with 5% (vol/vol) fetal bovine serum	100 μg/mL	Human epithelial-like HeLa cells in vitro	No effect on growth rate	[199]
0.5 DMSO pristine SWCNTs	25 μg/mL	Human embryo kidney (HEK 293) cells in vitro	G1 cell arrest and apoptosis	[200]

## Data Availability

Not applicable.
